# In vitro immunological and biological evaluations of the angiogenic potential of platelet-rich fibrin preparations: a standardized comparison with PRP preparations

**DOI:** 10.1186/s40729-015-0032-0

**Published:** 2015-11-27

**Authors:** Mito Kobayashi, Tomoyuki Kawase, Kazuhiro Okuda, Larry F. Wolff, Hiromasa Yoshie

**Affiliations:** 1 Division of Oral Bioengineering, Institute of Medicine and Dentistry, Niigata University, Niigata, Japan; 2 Division of Periodontology, Institute of Medicine and Dentistry, Niigata University, Niigata, Japan; 3 Advanced Research Center, The Nippon Dental University School of Life Dentistry at Niigata, Niigata, Japan; 4 Division of Periodontology, Department of Developmental and Surgical Sciences, University of Minnesota School of Dentistry, Minneapolis, MN USA

**Keywords:** Platelets, Fibrin, Plasma, Angiogenesis, Endothelial cells

## Abstract

**Background:**

Platelet-rich fibrin (PRF), a platelet-rich plasma (PRP) derivative mainly composed of fibrin networks, has been increasingly demonstrated to be effective in wound healing in clinical and pre-clinical animal studies. However, there has still been a concern that major growth factors may significantly be loss from PRF during its preparation through the slow clotting process. To address this concern, we compared the angiogenic potential of PRF and PRP by standardization of procedures based on volume ratios.

**Methods:**

PRP, PRF, and platelet-poor plasma (PPP) were prepared from the peripheral blood of healthy donors. PRF preparations were squeezed or homogenized to produce exudate (PRF*exu*) or extract (PRF*ext*), respectively. Concentrations of the angiogenic factors and their bioactivities were determined using ELISA kits, a scratch assay using endothelial cells and a chicken chorioallantoic membrane (CAM) assay.

**Results:**

In PRP and PRF preparations, both VEGF and PDGF-BB were significantly more concentrated than PPP. In the scratch assay, PRF*exu* and PRF*ext* were the most effective for wound closure. In the CAM assay, PRF membranes were the most effective for neovascularization.

**Conclusions:**

It is suggested that PRF preparations efficiently preserve the angiogenic factors and function not only as a scaffolding material but as a reservoir of angiogenic factors in wound healing.

## Background

Since Marx’s publications [1, 2], platelet-rich plasma (PRP) has been widely applied in regenerative therapy. However, because the preparation protocol is relatively complex and not standardized between laboratories, its clinical outcomes have often varied significantly among individual clinical research groups [3]. To overcome this disadvantage, Choukroun and his co-workers developed platelet-rich fibrin (PRF) by modifying the process of PRP preparation [3–5]. PRF can be prepared solely through the activation of an endogenous coagulation process without the aid of animal-derived coagulants such as bovine thrombin. Its advantages include operator-friendly preparation procedures and doctor-friendly handling when used in a clinical setting. However, the primary and more important advantage of PRF should be attributed to its clinical effectiveness rather than efficiency in preparation and handling.

Because PRF is mainly composed of fibrin fibers, it has been generally thought that PRF preparations must be distinguished from PRP, a “cocktail” of growth factors. Furthermore, there has been a concern that most growth factors may diffuse and be loss during the PRF preparation process. To address this concern, clinical and pre-clinical animal studies have increasingly been performed over the past 2 years [6–16]. The majority of published articles to date have concluded that PRF preparations were more effective or equally effective to PRP preparations in wound healing and tissue regeneration. These findings are not surprising because fibrin fibers would be expected to function as an efficient carrier to form a delivery system of growth factors [17]; however, the methods for PRF preparation and application are often not fully disclosed or vary across individual studies. Therefore, for a more precise comparison, it is necessary to standardize the procedures for preparation of PRF and to normalize their effectiveness by utilizing known volumes of blood samples.

For example, to standardize the handling of PRF exudate (PRF*exu*), in a previous study [18], we developed a compression device and proposed the use of this device to create a PRF membrane with uniform thickness and to minimize loss of PRF*exu* assuming that PRF*exu* contains significant amounts of important growth factors for wound healing. As a result of this comparative study using an ex vivo chick embryo chorioallantoic membrane (CAM) assay, it turned out that growth factors were distributed both in the exudate portion and in the fibrin network. Therefore, to more precisely compare the PRF with PRP preparations at the cellular and molecular levels, it is necessary to standardize the preparation procedures and to evaluate their potency by the volume ratios of the originally collected peripheral blood samples with the resulting preparation samples.

In this study, because the primary site of PRP action in wound healing and tissue regeneration is the formation of blood vessels [17, 19], we focused on vascular endothelial growth factor (VEGF) and its target action of angiogenesis and compared the concentrations of PRP and PRF preparations as to their biological effectiveness using ELISA and bioassay systems after performing the appropriate normalization.

## Methods

### Preparation and clotting of PRP

As previously described [20, 21], blood was collected from three healthy and non-smoking volunteers aged 28, 30, and 54 years (two females; one male), and PRP was prepared along with platelet-poor plasma (PPP) using the two-step centrifugation protocol. The number of platelets in the freshly prepared PRP and PPP samples was determined using an automated hematology analyzer (pocH 100iV diff: Sysmex, Kobe, Japan). Then, PRP and PPP preparations were frozen and stored at −20 °C until further used (usually within 2 weeks).

**Fig. 1:**
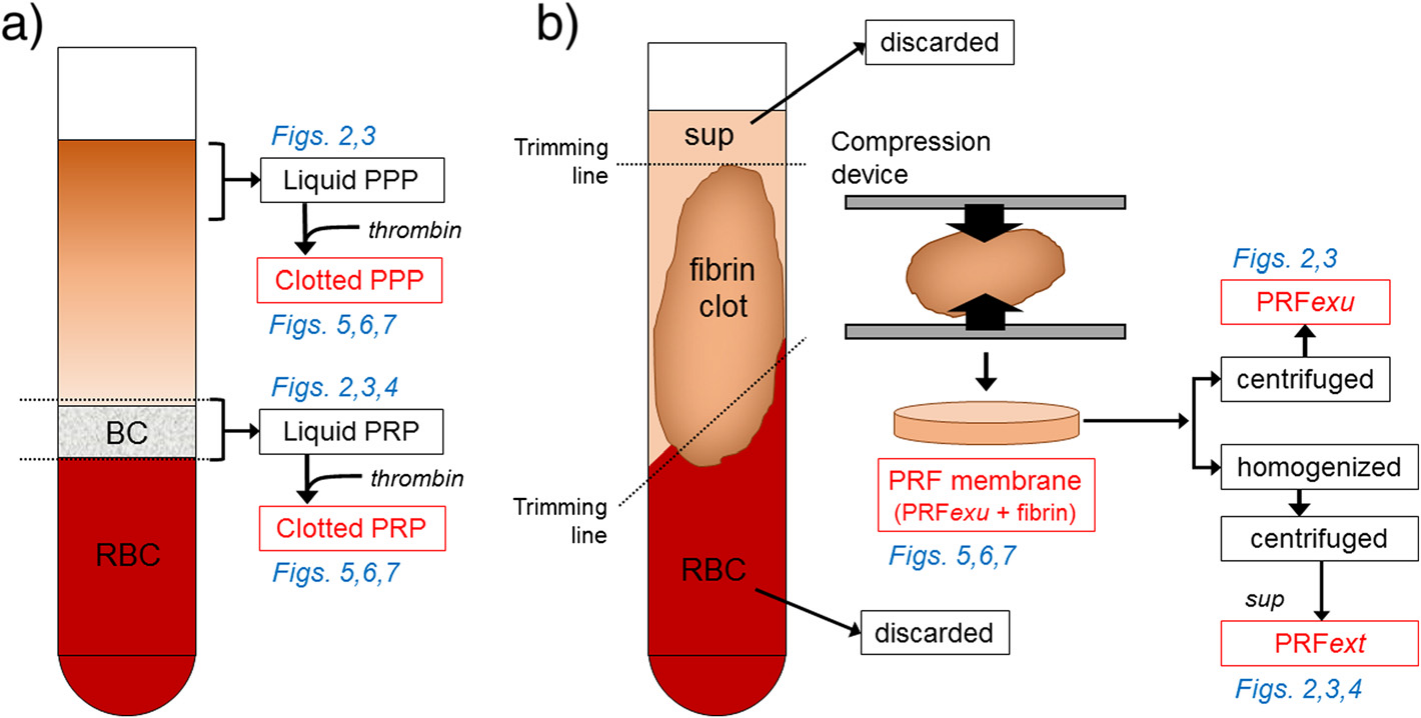
**a** Preparation of PPP and PRP samples. **b** Preparations of PRF samples (PRF*exu* and PRF*ext*)

Preparation of PPP and PRP samples is shown in Fig. 1a. For clotting, bovine thrombin (180 U) (Haematologic Technologies, Inc., Essex Junction, VT, USA) and 10 % CaCl_2_ (30 μL) were added to the indicated volumes of PRP or PPP preparations to form 5 × 5 × 1 mm (width × length × thickness) membranes. The relative volumes of individual preparations against the original blood samples were calculated and are summarized in Table 1.

**Table 1:** Comparison of sample volumes and preparation volumes

	Platelet-poor plasma (PPP)	PPP clot	Platelet-rich plasma (PRP)	PRP clot	Platelet-rich fibrin exudate (PRF*exu*)	Platelet-rich fibrin extract (PRF*ext*)	Platelet-rich fibrin (PRF) membrane
Starting blood sample volume (mL)	10^a^	10^a^	10^a^	10^a^	10	10	10
Resulting sample volume (mL) (vs. starting volume)	2.5 (1:4)		1 (1:10)		1 (1:10)	1 (1:10)	1^b^ (1:10)
Relative condensation (fold)	1		2.5		2.5	2.5	
Volume needed to mold a clot (mL) (vs. resulting sample volume)		0.25 (1:10)		0.2 (1:5)			
Number of 5 × 5 × 1-mm membrane		10^c^		5^c^			5–7^d^
Relative condensation (fold)		1		2			1.4–2.0

^a^This sample volume does not include the volume of acid citrate dextrose solution (ACD; 1.5 mL)

^b^(Acquired sample volume) = (blood sample volume) − (RBC fraction) − (serum volume)

^c^Estimated number of membranes

^d^Acquired number of membranes

The study design and consent forms for all procedures performed with the study subjects were approved by the ethical committee for human subject use at Niigata University Medical and Dental Hospital in accordance with the Helsinki Declaration of 1975 and as revised in 2008.

### Preparation, compression, and homogenization of PRF

As described previously [18], blood was collected from the same donors and immediately centrifuged by a Medifuge centrifugation system (Silfradent S. r. l., Santa Sofia, Italy). Preparation of PRF samples, PRF*exu* and PRF*ext* are shown in Fig. 1b. After eliminating the red thrombus, the resulting PRF preparations were compressed by the PRF compression device [18]. As described earlier, the stainless steel PRF compression device developed for PRF membrane preparation was composed of two spoon shaped parts. The clearance between the spoon parts when compressed was adjusted to be 1 mm. Thus, when the PRF clots were compressed with this device, a standard 1-mm thick PRF membrane was consistently prepared.

After compression, PRF membranes were centrifuged to squeeze out the PRF*exu*. Alternatively, PRF membranes were minced and homogenized on ice for 1 min with a micro-Multimixer (Ieda Trading, Corp., Tokyo, Japan) and centrifuged to obtain supernatants (PRF*ext*) (Fig. 1b). The resulting PRF*exu* and PRF*ext* were directly used along with PRP and PPP preparations for ELISA and the bioassays. Comparison of sample and preparation volumes is shown in Table 1. PPP, PRP, and PRF membrane preparations were standardized by preparing them from the same volume (10 mL) of blood samples.

### Determination of growth factor levels by ELISA

The concentrations of VEGF, PDGF-BB, and soluble DLL1 in frozen PPP, PRP, and PRF samples were determined using human VEGF, PDGF-BB, and DLL1 Quantikine ELISA Kits (R&D Systems, Inc., Minneapolis, MN, USA).

### Cell culture and scratch assay

Human umbilical vein endothelial cells (HUVECs) were obtained from Cell Applications, Inc., (San Diego, CA, USA) and cultured with HuMedia-EB2 supplemented with growth factors (Kurabo, Tokyo, Japan). For the scratch assay, the cells were seeded into 6-well plates at a density of 1 × 10^5^ cells/well and cultured in a CO_2_ incubator until they reached early confluence. The medium was then replaced with HuMedia-EB2 supplemented with reduced amounts of growth factors (1:5 in dilution). The monolayer was scratched using a scraper with a 1-mm blade and incubated for an additional 24 h with 2 % (*v*/*v*) PRF*exu*, PRF*ext*, PRP, or PPP. The wound areas were photographed at specific time points, and the width of the scratched gap was determined using ImageJ (National Institutes of Health, Bethesda, MD, USA).

### Western blotting analysis

HUVECs were seeded onto 6-well plates at a density of 1 × 10^4^ cells/well and pre-cultured for 2 days to form subconfluent cultures. After washing, the cells were treated with PPP, PRP, or PRF*ext* in a CO_2_ incubator (5 % CO_2_) for 10 min in serum-free Hank’s balanced saline solution (HBSS). After washing twice with ice cold PBS, the cells were lysed with Laemmli sample buffer as previously described [22]. Protein samples were fractionated using 10 % SDS-PAGE (ATTO, Tokyo, Japan) and electro-blotted onto PVDF membranes using the Trans-Blot® Turbo™ Transfer System (Bio-Rad Laboratories, Hercules, CA, USA).

After blocking with diluted Block A (1:2) (DS Pharma, Osaka, Japan) or 5 % BSA (Fraction V) (Sigma, St. Louis, MO, USA) in 0.1 % Tween 20-containing TBS (T-TBS) for 4–5 h at 4 °C, the membranes were probed overnight at 4 °C with the following primary antibodies: rabbit polyclonal anti-phospho-VEGFR2 (Y996) (1:2000 in dilution) (Cell Signaling Technology, Danvers, MA, USA), rabbit polyclonal anti-VEGFR2 (D5B1) (1:2,000) (Cell Signaling Technology), or goat polyclonal anti-actin antibody (1:1,000) (Santa Cruz Biotechnology, Inc., Santa Cruz, CA, USA). After washing three times with T-TBS, the membranes were probed with horseradish peroxidase-conjugated goat polyclonal anti-rabbit IgG H&L (1:5,000) (Abcam, Cambridge, MA, USA) or horseradish peroxidase-conjugated donkey anti-goat IgG (Santa Cruz Biotechnology) for 45 min at 4 °C. After washing, images were visualized using Clarity™ Western ECL Substrate (Bio-Rad) and imaged using a cooled CCD camera system (Image Capture; ATTO, Tokyo, Japan).

### The ex vivo chorioallantoic membrane model

As described previously [18], fertilized chicken eggs were incubated in a hatching incubator equipped with an automatic rotator (KingSURO20; Belbird, Siki, Japan) at 37 °C with a relative air humidity of 65 %. On embryo developmental day 11, a hole approximately 16 mm in diameter was opened in the eggshell, and clots of PPP and PRP and PRF membranes (5 × 5 mm) were placed on the central area of the CAMs. After the holes were sealed, the eggs were incubated for an additional 3 days. The CAM vasculature was macroscopically photographed at the initial time point (day 0) and the end point (day 3).

The number of vessels in the center circle (1 cm^2^) was determined using image analysis software (WinROOF, Mitani Corp., Fukui, Japan). In brief, RGB (red-green-blue) channel layers were separated, and the blue channel layer was manually adjusted to its threshold. After the images were binarized to clearly show blood vessels [23], the number of vessels was counted manually per cm^2^.

### Histological and immunohistochemical examination of CAM

After counting the number of blood vessels, CAMs were harvested, fixed, and embedded in paraffin for histological examination. As described previously [24], deparaffinized sections were subjected to hematoxylin-eosin (HE) staining and Masson’s trichrome staining.

Alternatively, the sections were antigen-retrieved and blocked with 2.5 % normal horse serum (Vector Labs, Burlingame, CA) and subsequently probed with a mouse monoclonal anti-α-smooth muscle actin (α-SMA) antibody (1:100) (Abcam, Cambridge, MA, USA) overnight at 4 °C, followed by incubation in the ImmPRESS® anti-mouse IgG (Vector). Immunoreactive proteins were visualized by a DAB substrate solution (Kirkegaard & Perry Laboratories, Inc., Gaithersburg, MD).

The number of α-SMA^+^ vessel-like structure (shown by arrows) inside the CAM was determined using image analysis software (WinROOF, Mitani Corp., Fukui, Japan). In brief, after the contrast of the images was enhanced manually, the number of vessels was counted. The area of the CAM in the cross-sections was also evaluated. Then, the number of vessels was normalized by utilizing the ratio of the area to the number of vessels per unit square (mm^2^).

### Immunofluorescence examination

HUVECs were seeded onto glass coverslips and pre-cultured for 24 h to form subconfluent cultures. The cells were treated with 2 % PRP or PRF*ext* for up to 3 h. The cells were then fixed and treated with FITC-conjugated mouse monoclonal anti-CD41 (1:5) (Beckman Coulter, Inc., Brea, CA, USA) or FITC-conjugated rabbit polyclonal anti-human fibrinogen (1:20) (Medical & Biological Laboratories Co., Ltd, Nagoya, Japan) for 1 h at 4 °C, as described previously [25]. After three washes with T-PBS, the cells were mounted with VECTASHIELD Mounting Medium with DAPI (Vector Laboratories, Burlingame, CA, USA) and examined using a fluorescence microscope (Nikon, Tokyo, Japan).

### Statistical analysis

The data were reported as the mean value ± standard deviation (S.D.). For multi-group comparisons, statistical analyses were performed to compare the mean values using one-way analysis of variance (ANOVA) followed by Tukey’s multiple comparison test (SigmaPlot 12.5; Systat Software, Inc., San Jose, CA, USA). *P* values <0.05 were considered significant.

## Results

**Fig. 2:**
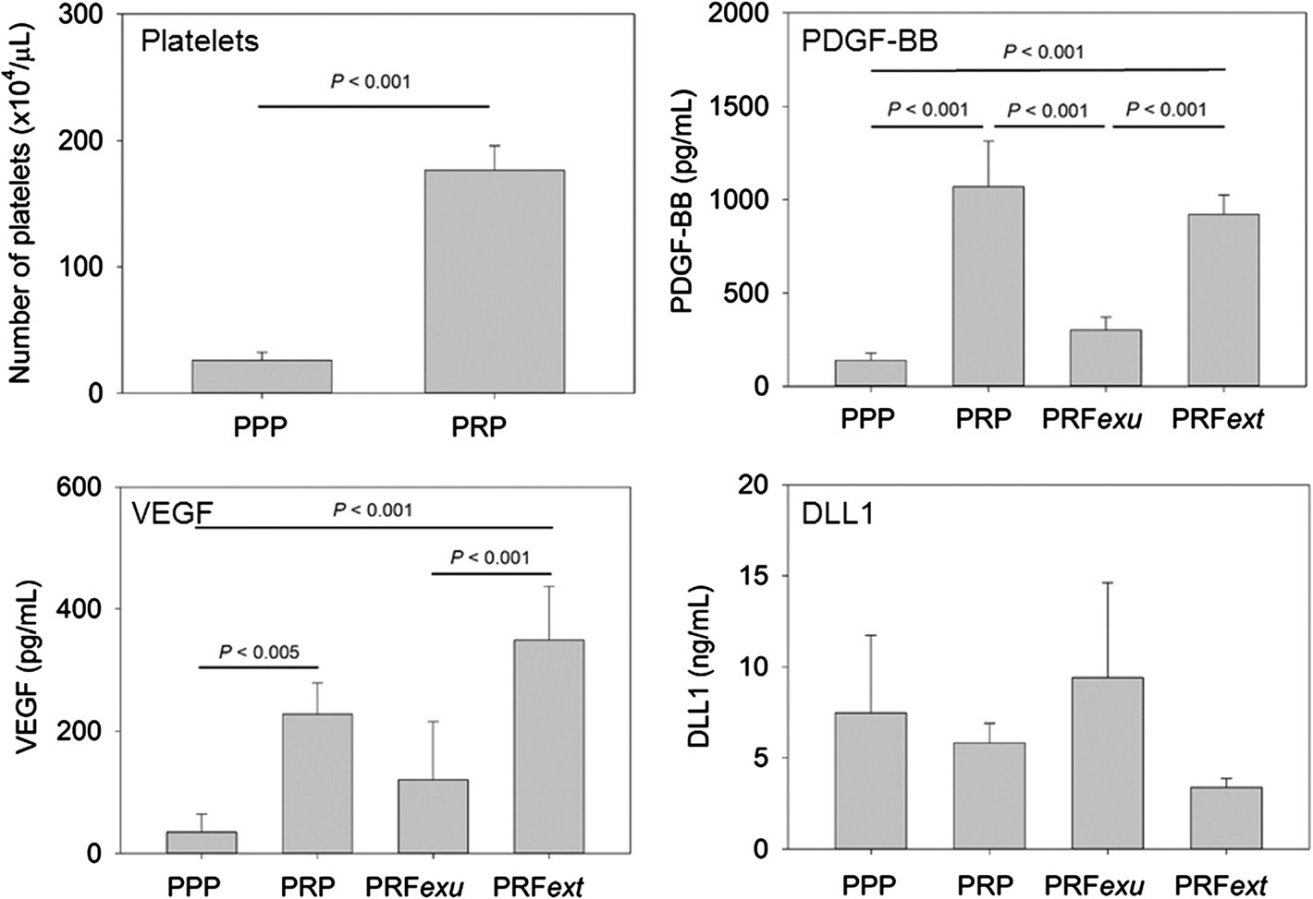
The concentration of platelets, VEGF, PDGF-BB, and DLL1 in PPP, PRP, PRF*exu,* and PRF*ext* preparations. *n* = 5

The concentration of platelets and growth factors in PPP, PRP, and PRF preparations are shown in Fig. 2. In PRP preparations, platelets were concentrated approximately 6.8-fold over PPP preparations. Although it is challenging to accurately count platelets in PRF preparations, we previously observed by SEM examination that platelets aggregated and attached to the surface of the PRF membrane, suggesting that platelets were concentrated on PRF membrane surfaces [18]. In support of this finding, PDGF-BB, a platelet-specific growth factor, was concentrated 7.6 and 6.5-fold in PRP and PRF*ext* preparations, respectively, when compared with PPP preparations. The potent angiogenic factors, VEGF and soluble DLL1, demonstrated contrasting results. VEGF was concentrated in PRP and PRF*ext* preparations 6.5 and 10.0-fold, respectively, when compared with PPP preparations, whereas soluble DLL1 was not concentrated in either PRP or PRF*ext* when compared with PPP. In addition, the concentration of another angiogenic factor, bFGF, was too low to reproducibly be detected in any of the preparations tested in this study (data not shown).

**Fig. 3:**
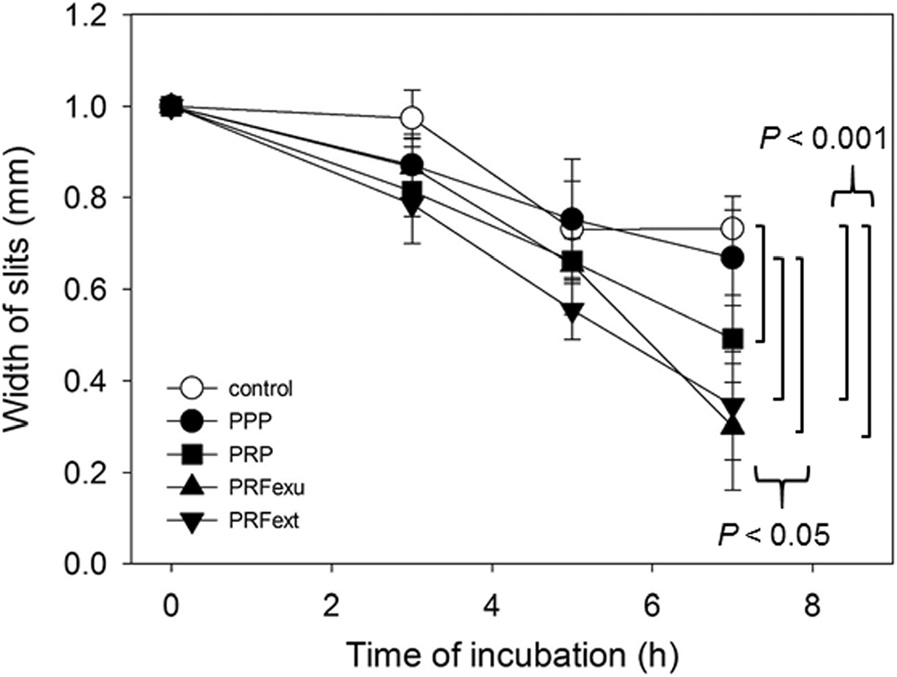
The time-course effects of PPP, PRP, PRF*exu,* or PRF*ext* preparations on the wound prepared in HUVEC cultures. The controls included no plasma-derived supplements. *n* = 5

The time-course effects of PPP, PRP, and PRF preparations on simulated wounds prepared in HUVEC confluent cultures are shown in Fig. 3. The wound closure was facilitated by PRP (2 %) and PRF preparations (2 %), but not PPP preparations (2 %). The order of potency was PRF*exu* ≥ PRF*ext* > PRP > > PPP at 7 h of incubation. The statistical significances are *P* < 0.05 (PPP vs. PRF*exu; PPP vs. PRFext; control vs. PPP*) and *P* < 0.001 (control vs. PRF*ext*; control vs. PRF*exu*). The controls included no plasma-derived supplements.

**Fig. 4:**
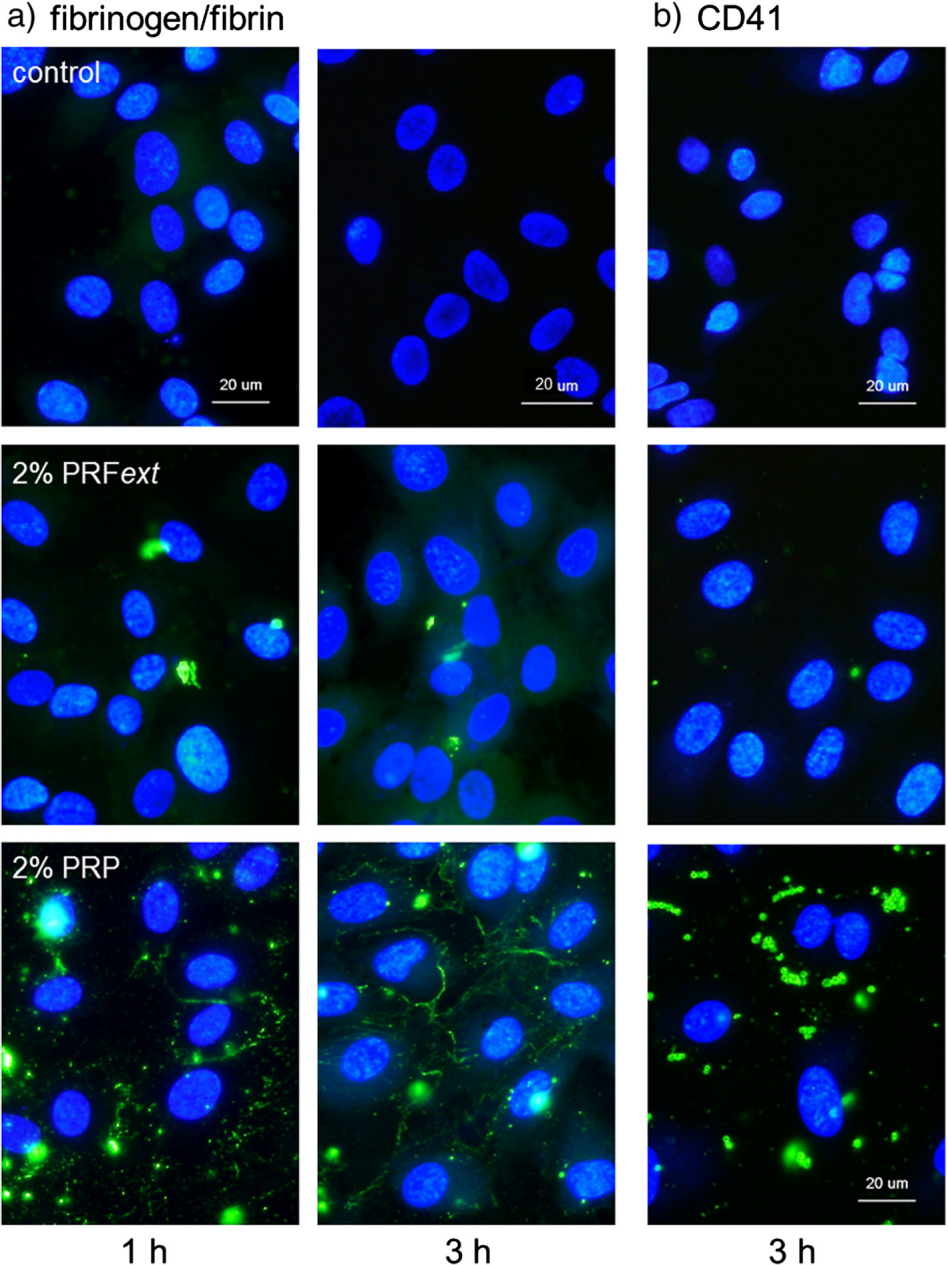
Immunofluorescence examination of fibrin fiber formation (**a**) and CD41^+^ platelet distribution (**b**) in PRP-treated HUVEC cultures. HUVECs were treated with 2 % PRFext- or 2 % PRP-containing media for up to 3 h. The cells were fixed and subjected to immunofluorescence examination. Similar data were obtained from two additional independent experiments (*n* = 3). Bar = 20 μm

The time-course effects of PRF*ext* and PRP on the formation of fibrin fibers in HUVEC cultures are shown in Fig. 4a. Typical fibrin fibers were formed time dependently only in PRP-added cultures, but not in controls without plasma-derived supplements or in PRF-added cultures. The distributions of CD41^+^ platelets in PRP-treated HUVEC cultures are shown in Fig. 4b. Significant numbers of CD41^+^ particles were found in PRP-added cultures, but not in the control cultures without plasma-derived supplements. A low number of CD41^+^ particles were found in PRF-added cultures.

**Fig. 5:**
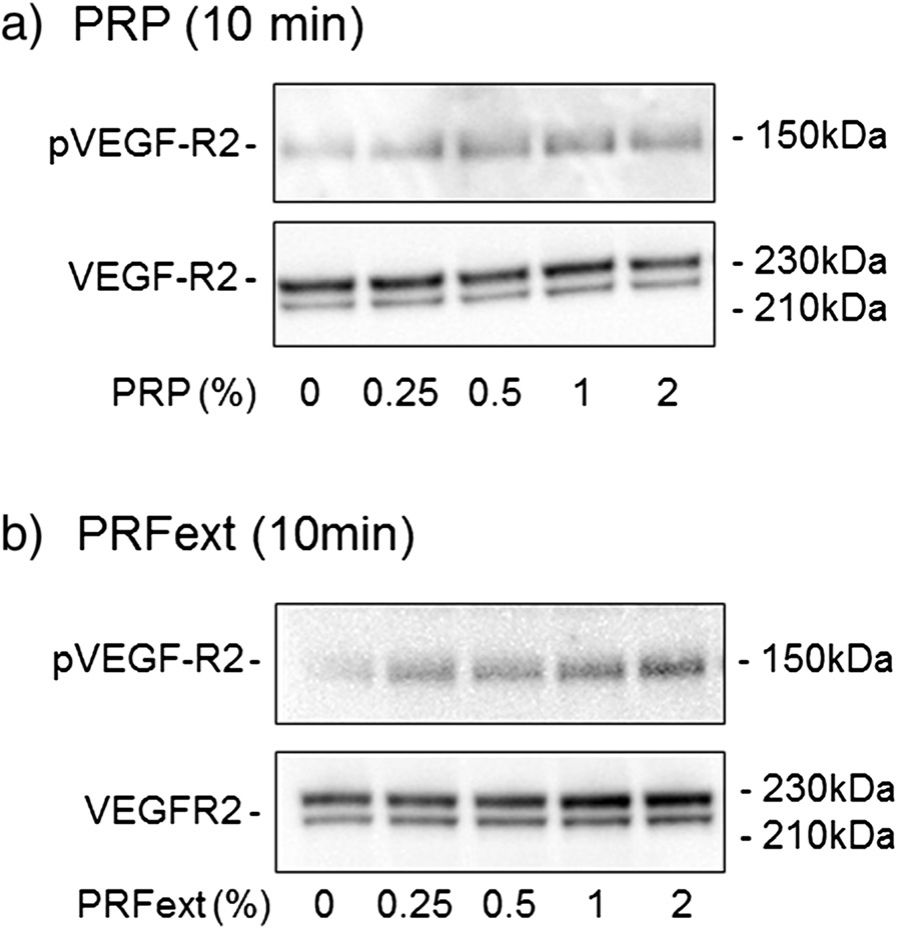
The dose-dependent effects of PRP (**a**) and PRF*ext* preparations (**b**) on phosphorylation of VEGFR2 in HUVEC at 10 min. Similar data were obtained from three additional independent experiments (*n* = 4)

The dose-dependent effects of PRP and PRF*ext* preparations on the phosphorylation of VEGFR2 are shown in Fig. 5. PRP and PRF preparations (0.25–2 %) dose-dependently phosphorylated VEGFR2 when compared with pan VEGFR2, and the potency of PRF*ext* preparations were similar to that of PRP preparations.

**Fig. 6:**
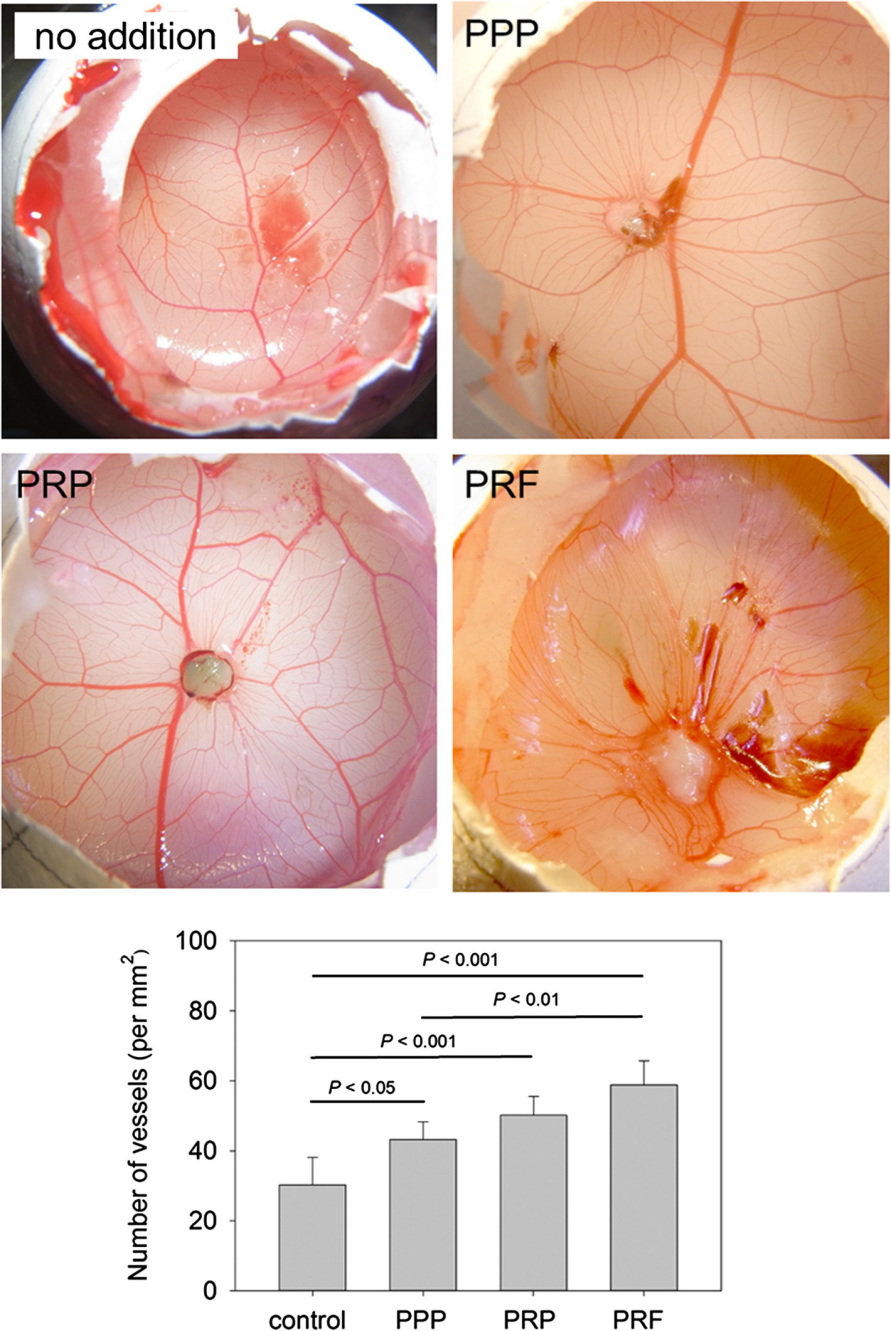
The effects of clotted PPP, clotted PRP, and PRF membrane preparations on new blood vessel formation in the CAM assay. The controls included no plasma-derived supplements. *n* = 5

The effects of clotted PPP, clotted PRP, and PRF membrane preparations on new blood capillary formation in the CAM assay are shown in Fig. 6. New capillary formation at 3 days after application was macroscopically examined. The image analysis for quantitation demonstrated that both PRP and PRF preparations significantly increased the number of blood capillaries. The order of potency was PRF ≥ PRP ≥ PPP and the statistical significances are *P* < 0.05 (control vs. PPP), *P* < 0.01 (PPP vs. PRF), and *P* < 0.001 (control vs. PRP; control vs. PRF). The controls included no plasma-derived supplements.

**Fig. 7:**
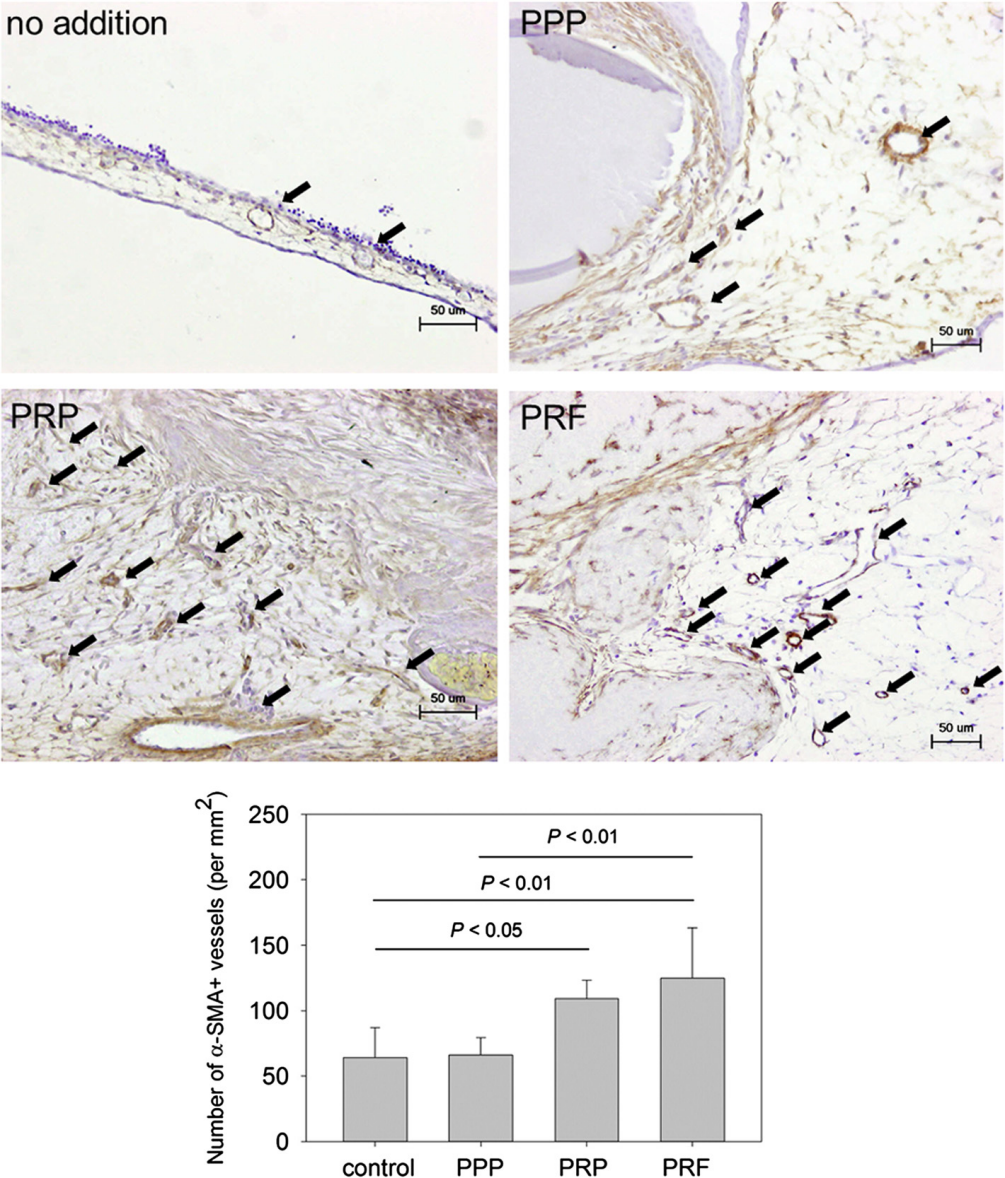
Immunohistochemical examination of the effects of clotted PPP, clotted PRP, and PRF membrane preparations on formation of α-SMA^+^ matured blood vessels in the CAM. Representative photomicrographs of α-SMA^+^ matured blood vessels (indicated by *arrows*). The controls included no plasma-derived supplements. *n* = 5

These effects were further confirmed by determining the number of mature blood vessels based on the concept that α-SMA is a marker of vascular smooth muscle cells. Alpha-SMA was immunohistochemically evaluated to determine the number of blood vessels. Immunohistochemical examination of the effects of clotted PPP, clotted PRP, and PRF membrane preparations on formation of α-SMA^+^ mature blood vessels in the CAM. As earlier demonstrated in the macroscopic examination of new capillary formation (Fig. 6), both PRP and PRF preparations significantly increased the number of mature blood vessels (Fig. 7). Here again, a similar trend of α-SMA-staining potency for estimating the number of mature blood vessels was PRF ≥ PRP > PPP. The statistical significances were *P* < 0.05 (control vs. PRP) and *P* < 0.01 (control vs. PRF; PPP vs. PRF). The controls included no plasma-derived supplements.

**Fig. 8:**
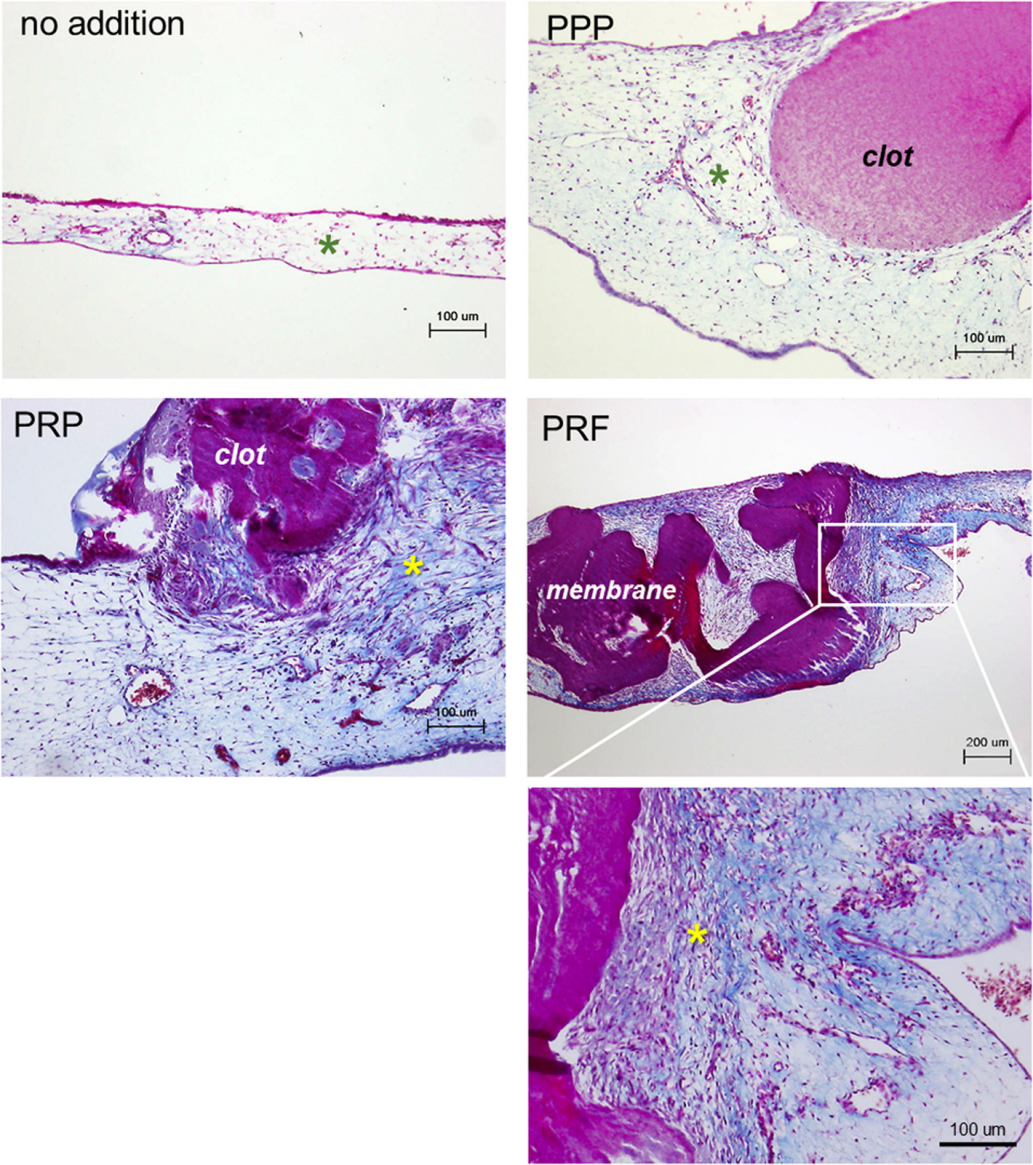
Histological examination of the effects of clotted PPP, clotted PRP, and PRF membrane preparations on the thickness and the structure of the CAM. Representative photomicrographs of Masson’s trichrome staining (*n* = 5). Cell nuclei and collagen are stained red and blue, respectively. *Asterisks* represent the regions for evaluation of cell density and collagen deposition. The controls included no plasma-derived supplements

Further histological examination with Masson’s trichrome staining was performed to examine the changes taking place in the CAM assay. The effects of clotted PPP, clotted PRP, and PRF membrane preparations on the thickness and the structure of the CAM are shown in Fig. 8. These preparations all significantly thickened the CAM by stimulating fibroblast proliferation and extracellular matrix (ECM) deposition. However, both PRP and PRF preparations were more potent at stimulating fibroblast proliferation and collagen deposition than PPP preparations. The controls included no plasma-derived supplements.

## Discussion

The PRF preparation procedure presented here is simple and less technique-sensitive than previously reported; however, it cannot be ruled out that, because of slow clotting, the centrifugation process possibly activates platelets to release growth factors more than what was predicted. It has widely been thought; however, without clear evidence, that even though they function as a scaffolding material, PRF preparations may not provide growth factors to the level that will synergistically facilitate wound healing and tissue regeneration. Dohan Ehrenfest and co-workers first reported that PRF preparations contain significant amounts of growth factors [5], and we independently demonstrated that the growth factors are distributed not only in PRF*exu* but also on and among fibrin fibers [18] (Kawase et al., unpublished observations). Therefore, evidence supports the notion that PRF preparations contain and deliver growth factors to the site of wound-healing.

Furthermore, it has recently been demonstrated in clinical and pre-clinical animal studies that PRF preparations have the potential of tissue regeneration at levels almost identical to or even greater than PRP preparations [6–16]. However, the majority of these results were obtained from macroscopic and/or histological examinations and therefore, these studies were phenomenological and did not investigate the mechanism or the mode of PRF action. To our knowledge, the potency and efficacy of PRP and PRF preparations have not precisely been compared with each other at the cellular and molecular levels. One of the possible reasons is that clinicians require evidence to support their clinical use of PRF. From a more experimental point of view, the lack of appropriate standardization or normalization methods between laboratories has hindered the comparison of PRP with PRF in wound-healing investigations. In this study, individual samples were normalized based on the volume ratio of the original whole blood samples to the resulting products (Table 1). It may not be the most suitable for quantitative comparison; however, we believe that the data we obtained will support or explain the previously published data [26–28].

In our study, the ELISA results of the PDGF-BB and VEGF, representative growth factors stored in platelets, demonstrated that platelets were highly concentrated in PRF preparations as well as in PRP preparations. In bioassays, PRF preparations exerted significant effects on wound closure and neovascularization at levels somewhat greater than PRP preparations. We speculate that a factor(s) existing in PRP, but not in PRF preparations, may attenuate the effect of PRP. The most plausible candidate is fibrinogen/fibrin because insoluble fibrin functions not only as a scaffolding material for many cell types but also as a carrier of growth factors [18, 29]. In previous studies [30] (Kawase et al., unpublished observations), we observed that fibrin fibers were formed by the addition of PRP preparations within 30 min and grown thereafter in human periodontal ligament cell cultures and osteoblastic MG63 cell cultures. In this study, an immunofluorescence examination demonstrated that fibrin fibers were similarly formed in HUVEC cultures within 60 min of PRP application, but not PRF*ext* application, and grown over time. Therefore, it is plausible that many growth factors can be trapped by the newly formed fibrin fibers.

Furthermore, it was demonstrated that many CD41^+^ particles, i.e., platelets and/or their membrane debris, were present in HUVEC cultures treated with PRP preparations, but not PRF*ext* preparations. It is known that endothelial cells bind to platelets through integrin α_v_β_3_ and CD40 [31]. Activation of integrin α_v_β_3_ facilitates cell migration [32], whereas activation of CD40 inhibits cell migration [33]. In our case, activation of CD40 was probably more dominant than the integrin to reduce HUVEC migration.

When the two PRF preparation forms, i.e., PRF*exu* and PRF*ext*, were compared by ELISA, both PDGF-BB and VEGF were contained at significantly higher levels in PRF*ext* than in PRF*exu*. However, the bioassay using the HUVEC scratch assay demonstrated that the potencies of the PRF*ext* and PRF*exu* were almost identical. At present, we do not have strong evidence to explain the discrepancy between the concentrations evaluated by ELISA and the order of potency obtained in the scratch assay for PRF*ext* and PRF*exu*. In conjunction with the well-known fact that PRP preparations contain a variety of growth factors [17], it seems likely that PDGF and VEGF, even at lower concentrations in PRF*exu* may act synergistically with other growth factors, such as TGFβ, bFGF, EGF, and IGF-I, to exert the maximum effects on cell migration.

In a previous study [25], we demonstrated using Western blotting analysis that PRP stimulates VEGF receptor type 2 (VEGFR2) to accelerate endothelial cell motility and wound closure. In this study, we demonstrated that PRF*ext* acted on HUVEC and phosphorylated VEGFR2 in a dose-dependent manner that was almost identical to that of PRP. This finding is essentially consistent with the data obtained from the immunological determinations. In this study, we did not demonstrate the direct activation of PDGF receptors by PRF and PRP preparations. However, judging from the literature previously published [34], it is anticipated that PDGF and VEGF synergistically function to facilitate neovascularization during the wound healing process.

The CAM assay provided additional interesting as well as informative data. The CAM was thickened by all preparations, and both PRP and PRF preparations were more potent than PPP preparations. This phenomenon could be explained by the direct action of TGF-β and PDGF, in addition to the probable indirect action of VEGF, all of which are concentrated in both PRP and PRF preparations [17, 20, 35, 36] and capable of stimulating the proliferation of fibroblasts. Among them, TGF-β is stored in a latent form in the extracellular matrix [37] and functions as the most potent known growth factor involved in collagen production [38, 39]. According to a recent review article [38], it was suggested that VEGF, TGF-β, and PDGF provided by PRP or PRF preparations in high concentrations cooperatively induced dynamic reciprocal interactions between the cells and ECM to thicken the CAM. In addition, it turns out that this simple ex vivo experimental system can be applied in examining possible dynamic interactions between ECM and CAM.

## Conclusions

These findings suggest that the major angiogenic growth factors, such as PDGF and VEGF, are not significantly diffused away from platelets activated by endogenous thrombin during centrifugation but efficiently preserved in PRF preparations and that the angiogenic potential of PRF preparations is compatible with that of PRP preparations. In conjunction with the user-friendly preparation procedure and high-handling efficiency, we would recommend the clinical use of PRF preparations as a higher-quality, clinically effective substitute for PRP preparations in wound healing therapy.

## Abbreviations

ACD: acid citrate dextrose solution
ANOVA: one-way analysis of variance
CAM: chick embryo chorioallantoic membrane
DLL1: delta-like protein 1
ECM: extracellular matrix
EGF: epidermal growth factor
ELISA: enzyme-linked immunosorbent assay
HE: hematoxylin-eosin
HUVEC: human umbilical vein endothelial cells
IGF-I: insulin-like growth factor-I
PDGF: platelet-derived growth factor
PPP: platelet-poor plasma
PRF: platelet-rich fibrin
PRF*ext*: platelet-rich fibrin extract
PRF*exu*: platelet-rich fibrin exudate
PRP: platelet-rich plasma
RGB: red-green-blue
TGFβ: transforming growth factor-β
T-TBS: 0.1 % tween 20-containing Tris-based saline
VEGF: vascular endothelial growth factor
VEGFR2: VEGF receptor type 2
α-SMA: α-smooth muscle actin
